# Raman Characterization on Two-Dimensional Materials-Based Thermoelectricity

**DOI:** 10.3390/molecules24010088

**Published:** 2018-12-27

**Authors:** Zuoyuan Dong, Hejun Xu, Fang Liang, Chen Luo, Chaolun Wang, Zi-Yu Cao, Xiao-Jia Chen, Jian Zhang, Xing Wu

**Affiliations:** 1 Shanghai Key Laboratory of Multidimensional Information Processing, Department of Electronic Engineering, East China Normal University, Shanghai 200241, China; 51181213021@stu.ecnu.edu.cn (Z.D.); 52151213012@stu.ecnu.edu.cn (H.X.); 52161213023@stu.ecnu.edu.cn (F.L.); 52161213022@stu.ecnu.edu.cn (C.L.); clwang@ee.ecnu.edu.cn (C.W.); 2 Center for High Pressure Science and Technology Advanced Research, Shanghai 201203, China; ziyu.cao@hpstar.ac.cn (Z.-Y.C.); xjchen@hpstar.ac.cn (X.-J.C.); 3 Shanghai Institute of Intelligent Electronics & Systems, Fudan University, Shanghai 200433, China

**Keywords:** Raman spectroscopy, two-dimensional materials, thermoelectricity, thermal conductivity, graphene

## Abstract

The emergence and development of two-dimensional (2D) materials has provided a new direction for enhancing the thermoelectric (TE) performance due to their unique structural, physical and chemical properties. However, the TE performance measurement of 2D materials is a long-standing challenge owing to the experimental difficulties of precise control in samples and high demand in apparatus. Until now, there is no universal methodology for measuring the dimensionless TE figure of merit (*ZT*) (the core parameter for evaluating TE performance) of 2D materials systematically in experiments. Raman spectroscopy, with its rapid and nondestructive properties for probing samples, is undoubtedly a powerful tool for characterizing 2D materials as it is known as a spectroscopic ‘Swiss-Army Knife’. Raman spectroscopy can be employed to measure the thermal conductivity of 2D materials and expected to be a systematic method in evaluating TE performance, boosting the development of thermoelectricity. In this review, thermoelectricity, 2D materials, and Raman techniques, as well as thermal conductivity measurements of 2D materials by Raman spectroscopy are introduced. The prospects of obtaining *ZT* and testing the TE performance of 2D materials by Raman spectroscopy in the future are also discussed.

## 1. Introduction

Thermoelectricity, which is a property of materials of directly realizing the transformation between heat and electric energy, has attracted extensive research interest due to the issues of severe energy wastage and insufficient resources affecting society. Since the Seebeck effect was discovered in 1821, the development of thermoelectricity is contingent on seeking and discovering new thermoelectric (TE) materials with a high figure of merit (*ZT*) [1,2,3,4]. It is imperative to identify novel TE materials or to develop the new methods to promote TE performance of known materials, realizing their wide applications in industry. In 2004, Novoselov and Geim successfully prepared single-layer graphene by a mechanical exfoliation method [5], which greatly stimulated studies on two-dimensional (2D) materials. Subsequently, layered molybdenum disulfide (MoS_2_), hexagonal boron nitride (h-BN), black phosphorus (BP), etc. were investigated, expanding the 2D materials family and promoting their development in electronics, photonics, mechanics, and thermotics [6,7,8,9,10,11,12]. Importantly, owing to their unique properties, 2D materials have attracted tremendous attention to thermoelectricity and become potential candidates for advancing TE performance [13,14,15,16,17,18,19,20]. However, it is difficult to exactly measure the TE performance of 2D materials due to their unique structure properties and a new measurement system applied for 2D materials is needed. 

Based on the discovery of the light scattering effect in 1928, Raman spectroscopy has become a versatile tool to characterize materials. Raman spectroscopy can provide both structural and electronic information of samples rapidly and non-destructively, being one of the most important techniques for studying 2D materials [21]. The thermal conductivity of materials is a critical parameter to evaluate the TE performance, whereas its measurement in 2D materials is a difficult task. Balandin et al. firstly employed Raman spectroscopy to measure the thermal conductivity of suspended single-layer graphene in 2008 [22], leading to a fruitful advancement in the experimental thermal studies of 2D materials. This method provides the possibility for systematic testing of TE properties, and is guiding the significance for the development of TE materials in the future. This review starts by introducing the background and advancement of thermoelectricity, briefly presenting the development of 2D TE materials in recent years. Then, we review the working principles and applications of Raman spectroscopy. Finally, several examples of how Raman spectroscopy can be used to measure the thermal conductivity of 2D materials are discussed in detail. Prospects, ideas and challenges for the comprehensive measurement of 2D TE performance in the future are reviewed as well.

## 2. Thermoelectric and 2D Materials

### 2.1. Background of Thermoelectricity

The TE effect comprises the Seebeck and the Peltier effects. In 1821, Seebeck found that the magnetic needle deflected in the presence of different connected metals under a temperature gradient. Essentially, the electric current was generated by different conductors under a temperature difference, and a magnetic field was generated by the current relying on the Oersted electromagnetism effect, which effected the deflection of the magnetic needle. 

**Figure 1:**
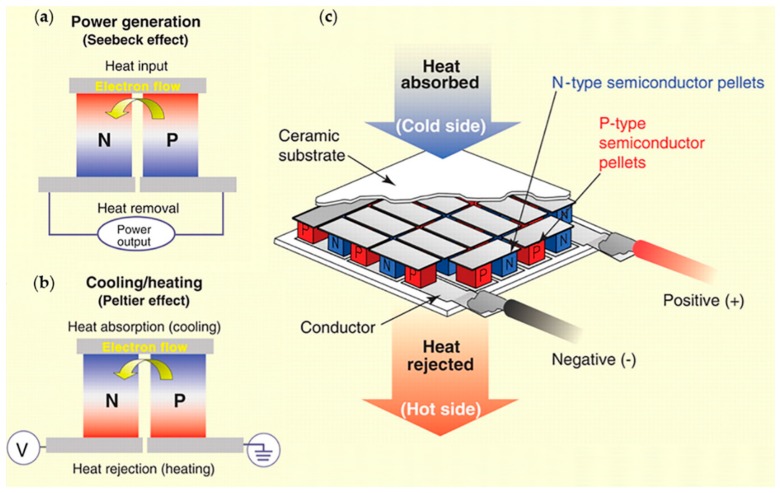
Illustration of TE effect and represented devices. (**a**) Schematic of the Seebeck effect. Electric current is produced when P-type and N-type semiconductors are placed under temperature differences at the same time. (**b**) Schematic of the Peltier effect. Heating or cooling is generated when electric current flows through P-type and N-type semiconductors. (**c**) Schematic of a TE generator. To improve the system-level conversion efficiency, P-type and N-type semiconductor pellets are connected in parallel under ceramic substrates, forming TE devices. Reproduced with permission from [2]. Copyright 2008, American Association for the Advancement of Science.

The phenomenon of generating electricity by employing a temperature difference is thus called the Seebeck effect. As shown in Figure 1a, if the N- and P-type semiconductors module at the top is the heat input and at the bottom is the heat removal part, carriers in semiconductors driven by the temperature difference move from the heat terminal to the cool terminal, producing an electron flow from the P-type semiconductor to the N-type semiconductor in the TE module. This is based on the Seebeck effect. The Peltier effect, observed by Peltier in 1834, is the inverse of the Seebeck effect. As shown in Figure 1b, heating or cooling is generated when a TE module is under a voltage, due to carriers taking away heat while moving under the electric field. Besides, the Thomson effect also exists, but can be neglected in this system. Thus, the TE effect mainly consists of the Seebeck and Peltier effects, which are the basis of TE devices. Figure 1c is the illustration of TE devices types, which are connected by large numbers of N- and P-type semiconductors modules in series to increase operating voltage and spread heat flow [2,3].

TE materials have extensive applications, mostly related to TE power generation and refrigeration technology. The former includes waste heat recovery, use of solar energy, and power supplying for wearable electronics [23,24,25,26]. The latter is widely applied in many areas of electric refrigeration due to its solid-state nature, absence of vibrations, simplicity and environmental friendliness [27].

The criteria of evaluating TE materials performance is the conversion efficiency between heat and electricity, which is directly related to the dimensionless TE figure of merit (*ZT*). For a generator, their relationship could be expressed as [1]:

(1) $$\eta =\frac{T_{h}-T_{c}}{T_{h}}\cdot \frac{\sqrt{1+ZT}-1}{\sqrt{1+ZT}+\frac{T_{c}}{T_{h}}}$$

where *η* is conversion efficiency, *T_h_* and *T_c_* are the temperature of heat and cool terminals, respectively. As it is shown, the generator efficiency depends on *ZT* strongly, thus optimizing *ZT* is a primary operation for enhancing TE performance. Before introducing the *ZT* value, Seebeck coefficient needs to be a simple explanation. The Seebeck coefficient is a parameter in Seebeck effect, which is a measure of the magnitude of the TE voltage generated per degree of temperature difference between two terminals of the semiconductor module. The definition is given below:

*S* = *dV*/*dT* (2)

where *S* is Seebeck coefficient, *V* is the TE voltage. *ZT* is defined as

*ZT* = *S*^2^*σT*/*κ* (3)

in which *κ* represents thermal conductivity and *σ* represents electrical conductivity. From this formula, it is obvious that *ZT* depends on the Seebeck coefficient, absolute temperature *T*, the electrical conductivity, and the thermal conductivity. However, *S*, *σ*, and *κ* are interrelated in traditional bulk TE materials, so it is very difficult to control these variables independently to enhance *ZT*. The interdependencies are mainly reflected in two aspects. One is between the Seebeck coefficient and the electrical conductivity in specific materials. For metals or degenerate semiconductors (parabolic band, energy-independent scattering approximation) the *S* is given by:

(4) $$S=\frac{8\pi^{2}k_{B}^{2}}{3eh^{2}}m^{*}T{\left(\frac{\pi }{3n}\right)}^{\frac{2}{3}}$$

and *σ* is expressed as:

*σ* = *neμ* (5)

where *k_B_* is Boltzmann constant, m* is the effective mass of the carrier, *n* is the carrier concentration, and *μ* is the carrier mobility. To ensure that the Seebeck coefficient is large at certain temperature, one way is to raise the effective mass, and the other is to reduce the carrier concentration. However, both are impracticable because both can reduce the electrical conductivity according to Equation (5). The other interdependency stems from the electrical conductivity and the thermal conductivity. The thermal conductivity mainly consists of two parts, which are *κ_e_* coming from electrons or holes transporting heat, and *κ_l_* coming from phonons traveling through the lattice, respectively. This can be expressed as:

*κ* = *κ_e_* + *κ_l_* (6)

According to Wiedemann–Franz law, the electrons’ thermal conductivity (*κ_e_*) is directly related to the electrical conductivity (*σ*):

*κ_e_* = *LσT* (7)

where *L* is the Lorenz factor that is a constant varying with carrier concentration. These clearly show enhancement on *ZT* is a tricky thing due to the coupled relationship of the thermal and the electrical conductivities [1].

The correlation and complexity between the parameters which are ubiquitous in conventional TE materials, increasing the difficulties of optimizing *ZT* and being the major causes limiting the development of thermoelectricity.

### 2.2. Development of TE Materials

Under the efforts of many scientific researchers around the world, the development of traditional bulk TE materials has made great progress [28,29,30]. Bismuth telluride (Bi_2_Te_3_) and its alloys are the classical TE materials with large Seebeck coefficients and are widely used in the commercial field [31,32,33,34]. Silicon germanium (SiGe) and its alloys are excellent TE materials suitable for high temperature applications, that are used in TE modules for deep-space missions to convert radio-isotope heat into electricity [35,36]. Lead telluride (PbTe) is also a perfect TE material studied by researchers all the time [37,38]. In addition, skutterudite [39,40], half-Heusler [41], clathrates [42], etc. also cause great interest and have become an indispensable part of the TE family. Other than these, SnSe was studied in depth by Zhao et al. [43,44] and Wei et al. [45] in recent years, becoming a promising TE material.

It is shown that most state-of-the-art TE materials have maximum *ZT* values between 1 and 2.5 [46]. Nevertheless, such a value is not enough to achieve large-scale application of thermoelectricity [47]. It is necessary to promote the TE property of materials, in which one intriguing way is to explore low-dimensional materials. The two interdependencies of parameters influencing *ZT* discussed above, in a way, can be decoupled in low-dimensional materials. The reasons can be explained as: (1) enhancing the Seebeck coefficient as well as controlling the Seebeck coefficient and electrical conductivity independently because of quantum-confinement effects, (2) increasing the ratio of electrical conductivity to thermal conductivity taking advantage of numerous interfaces to scatter phonons more effectively than electrons. Both theoretical studies and experimental results show that reducing the material dimensions can increase the *ZT* value of TE materials [48,49,50,51]. Low-dimensional materials are pointed out to provide a new direction on designing high-performance TE materials [33,52,53,54,55,56]. Low-dimensional materials include zero-dimensional (0D), such as quantum dots; one-dimensional (1D) ones, such as nanowires; and 2D materials, such as quantum wells and superlattices. This review mainly involves 2D materials.

### 2.3. 2D Materials

**Figure 2:**
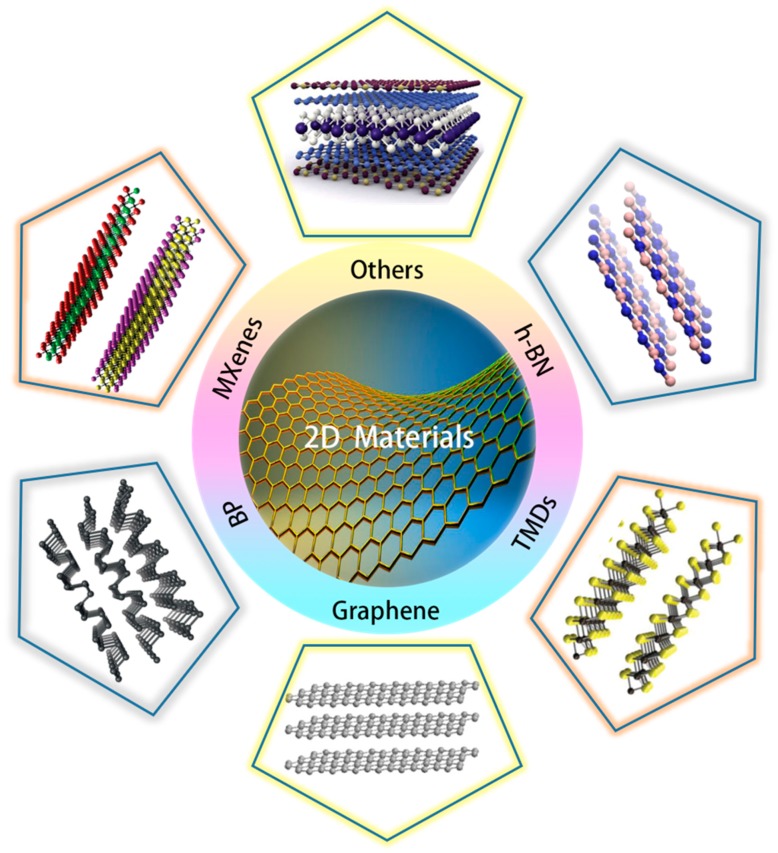
Schematic of 2D layered materials with their names. (Graphene) Reproduced with permission from [73]. Copyright 2014, American Chemical Society. (TMDs) Reproduced with permission from [74]. Copyright 2011, Nature Publishing Group. (h-BN) Reproduced with permission from [75]. Copyright 2016, Multidisciplinary Digital Publishing Institute (MDPI). (MXenes) Reproduced with permission from [76]. Copyright 2015, American Chemical Society. (BP) Reproduced with permission from [77]. Copyright 2014, Nature Publishing Group. (Others) Reproduced with permission from [78]. American Association for the Advancement of Science.

2D materials are crystalline materials consisting of single or a few layers of atoms with the lateral size being much larger than the thickness. The main categories of 2D materials are shown in Figure 2, which can help grasp and understand the 2D materials in a macroscopic way. Since the successful preparation of graphene, the employment of 2D materials has become a possibility, triggering a boom in the study of 2D materials. The continuous improvement of 2D material preparation technology [7] and manipulation of materials at the atomic scale [57,58], have greatly promoted the development of 2D materials and 2D devices [59,60,61,62]. 2D materials are widely applied in nanoelectronics and optoelectronics due to their outstanding optical, electronic, and other physical properties [63]. The multiplicity of the types and properties of 2D materials provide a broad space for the development of thermoelectricity. The two-dimensionalization of materials, for one thing depending on increasing the density of states near E_F_ (Fermi level) to enhance the Seebeck coefficient, for another using the interface scattering of phonons to reduce the thermal conductivity, is advantageous for the improvement of TE performance. In recent years, graphene [64,65], transition metal dichalcogenides (TMDs) [66,67,68,69,70], MXenes [15,71,72], etc. were found to possess excellent TE performance, paving the way for further exploration of 2D TE materials.

### 2.4. Advancement of 2D TE Materials

Advancement of 2D TE materials is the most representative example in term of the strategy of reducing the dimensionality to increase *ZT*. Thin-film materials as a class of 2D materials, are widely studied for their excellent TE performance and have made great progress in enhancing TE performance. In 2001, Venkatasubramanian et al. first reported on a Bi_2_Te_3_/Sb_2_Te_3_ superlattice film material prepared by metal organic chemical vapor deposition, which increases the *ZT* up to 2.4 [79]. Subsequently, Harman et al. used a molecular beam epitaxy technique to prepare a PbSe_0.98_Te_0.02_/PbTe quantum dot superlattice film with a *ZT* of 2.0 or higher at 300 K in 2002 [80]. Until now, superlattice films remain one of the most attractive research directions in the field of thermoelectrics. In 2017, Tian et al. presented an exfoliation-and-reassembly approach to produce a flexible N-type TiS_2_/organic superlattice film for low-temperature TE applications, which shows a high power factor (*S*^2^*σ*) after annealing under vacuum [81]. Al_2_O_3_/ZnO superlattice film [82] and V-telluride superlattice thin films [83] were reported in succession, and they show promising TE performance.

**Figure 3:**
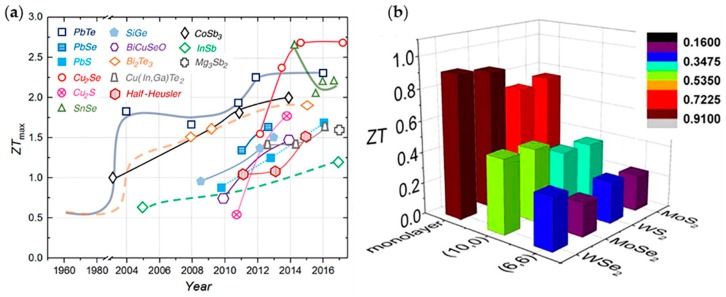
TE figure of merit (*ZT*) of materials. (**a**) Representation of the maximum *ZT* values of various 2D and traditional TE materials. Among them, SnSe [87], Cu_2_S [89], Cu_2_Se [90], and BiCuSeO [91] are investigated to have 2D or quasi-2D characteristics. Reproduced with permission from [46]. Copyright 2017, American Association for the Advancement of Science. (**b**) Comparison histogram of *ZT* values for diverse configuration of MoS_2_, WS_2_, MoSe_2_, and WSe_2_ studied by theoretical calculation. Here (10,0) and (6,6) are from the nomenclature representing armchair and zigzag single-wall Transition metal dichalcogenide nanotube (TMDNT). The different colors of columns refer to the extent of *ZT* value. From this histogram, one can see monolayer TMDs have a perfect *ZT* value clearly. Reproduced with permission from [67]. Copyright 2015, American Chemical Society.

Besides superlattice films, the development of layered 2D TE materials is also endless. Sharma et al. studied the TE properties of 2D telluride (Te) by first principles calculations and semiclassical Boltzmann transport theory, and the maximum *ZT* = 0.8 was achieved [13]. Monolayer PdSe_2_ was investigated by Qin et al. and its *ZT* reached 1.1 along the *x* direction for P-type doping at room temperature [84]. The TE properties of 2D InSe, SnSe, Pb_2_Se_3_, MoS_2_, and GeAs_2_ were also studied through theoretical and experimental studies in recent years [14,20,67,85,86,87,88]. As it is shown in Figure 3a, the development of TE materials has been rapid in recent years. In the theoretical category, *ZT* has reached and exceeded 2.5, and 2D materials such as SnSe, Cu_2_S and BiCuSeO have contributed greatly. 

Figure 3b is a comparison histogram of *ZT* for diverse TMD configurations, showing that monolayer TMDs acting the typical 2D TE materials, possess perfect TE performance with high *ZT*, and the maximum *ZT* is close to 1 [67]. Although the *ZT* values of 2D TE materials are currently still unable to reach that of the state-of-the-art nanocomposites and bulks, there are some advantages in 2D materials. (1) The interdependencies among the parameters of *ZT* in 2D materials can be decoupled to a certain degree discussed above, which is beneficial to optimize the *ZT* by decreasing the thermal conductivity due to phonon interface scattering. 2D TE materials have great room for improvement. (2) 2D materials are excellent for studying flexible, stretchable, and skin-friendly devices, which in conjunction with thermoelectricity, will facilitate the development of, for example, self-powered devices and TE sensors. (3) 2D TE materials enable the fabrication and application of miniaturized and integrated devices. It is worth mentioning that, the chip heat dissipation problem has become a major factor in limiting the development of integrated circuits (IC). 2D TE materials are able to be integrated into IC chips and take advantage of the waste heat supplying electricity for IC, accelerating the advancement of IC industry. All of the above signs indicate that the 2D TE materials are competitive candidates in the field of future thermoelectricity.

The *ZT* parameter for evaluating the performance of TE materials is conventionally obtained by measuring the thermal and electrical conductivities, the Seebeck coefficient of the TE material at a certain temperature. The measurement of the *ZT* in the bulk materials is relatively facile. Nevertheless, the test method of *ZT* for the film and the superlattice TE materials has higher requirements in terms of technology and sample preparation. Thus, a specially designed technique is required urgently. However, so far, a universal standard of systematically measuring *ZT* of 2D materials is still lacking owing to the experimental difficulties of precise control in samples and high demands on the apparatus. In 2018, Wang et al. summarized the instruments for measuring the Seebeck coefficient of thin-film thermoelectric materials [92]. For measuring thermal conductivity of 2D materials, the Raman spectroscopy technique is an effective method due to its non-destructive properties and simple operation. Besides, the time-domain thermoreflectance (TDTR) technique has also become a popular method in measuring thermal conductivity of films and micro-sized materials in recent years. They all have their own challenges and advantages. For instance, TDTR is an attractive approach to measuring the challenging interface thermal conductivity of dissimilar materials, and has an ultrafast measurement speed. Nevertheless, it has been challenging to measure thermal conductivity along different directions and its measurement process is relatively complex [93,94]. In contrast, the Raman method of measurement is much simpler.

## 3. Raman Technique

### 3.1. The Principles of Raman Spectroscopy

**Figure 4:**
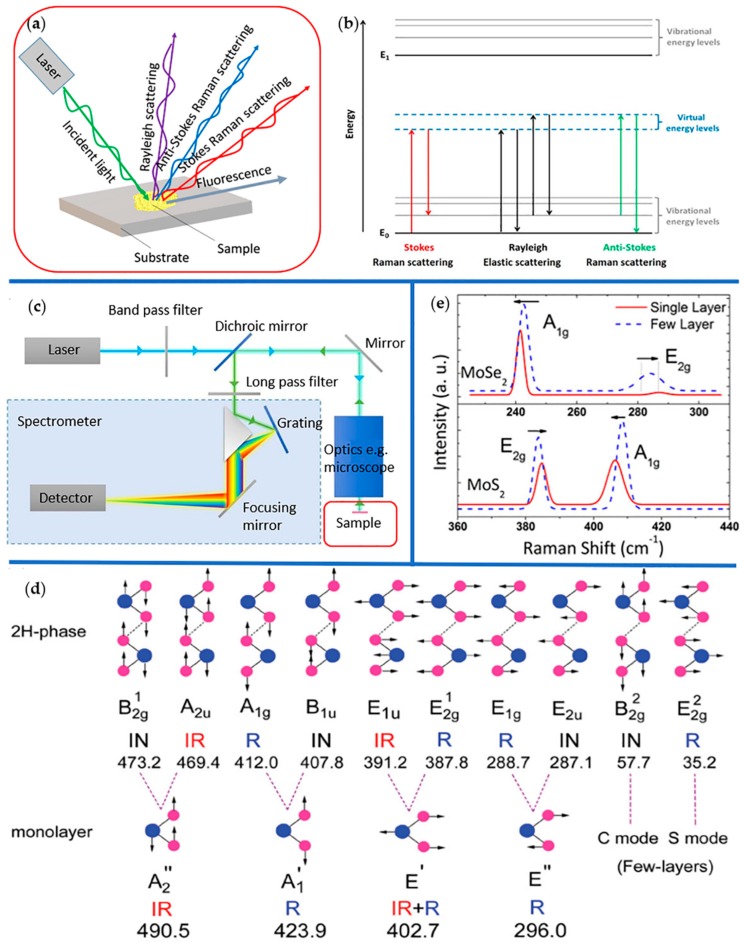
Raman spectroscopy and its production principle. (**a**) Illustration of the incident light (the green line) on a sample surface producing fluorescence (the gray line) and light scattering including Rayleigh scattering (the purple line), anti-Stokes Raman scattering (the blue line), and Stokes Raman scattering (the red line). Reproduced with permission from [96]. Copyright 2016, Nature Publishing Group. (**b**) Diagram of energetic transitions involved in Raman scattering. Top and bottom of the diagram are vibration energy levels, and the middle is virtual energy levels, respectively. Raman scattering is inelastic scattering produced by energy level transition of electrons excited by incident photons, which is divided into Stokes Raman scattering (the red icon) and anti-Stokes Raman scattering (the green icon) differencing from Rayleigh scattering (the black icon). In Stokes Raman scattering, the incident photon is higher frequency than the scattered photon, meaning that the incident photon has more energy, while in anti-Stokes scattering, the incident photon is of lower energy. (**c**) Typical setup of instrumentation within a spontaneous micro-Raman spectroscopic system. An incident light (represented by the blue line) emitted by laser source, passing through the band pass filter and the dichroic mirror, hits on the sample through the optical microscopy system. Then, the scattering light (represented by the green line) from the sample passes through the reflected mirror, dichroic mirror, and long pass filter in sequence, after that the light is dispersed by the spectrometer, collected by the detector finally. Reproduced with permission from Ref. [97]. Copyright 2017, Nature Publishing Group. (**d**) Optical vibration modes of 2H-MoS_2_ and monolayer MoS_2_. TMD (MX_2_) have three polytypes according to their lattice structure, including 1T-, 2H-, and 3R-polytype. Here the model diagram of 2H-polytype is presented, in which the lattice vibrational mode A_1_g corresponds to the out-of-plane relative motion of X atoms and E_2g_ corresponds to the in-plane opposing motion of M and X atoms. A_1g_ and E_2g_ of Raman spectrum are presented in the following diagram. Reproduced with permission from Ref. [98]. Copyright 2014, American Physical Society. (**e**) The Raman spectrum of single (solid red line) and few (more than 10) layers (dash blue line) MoSe_2_/MoS_2_. It can be clearly seen that both A_1g_ and E_2g_ modes are frequency shifted due to changes of the layer. Reproduced with permission from Ref. [99]. Copyright 2012, American Chemical Society.

Raman spectroscopy is a technique used to identify molecules, study chemical bonding, characterize microstructures of materials, measure thermal conductivity, etc. by measuring the frequency shift of inelastic scattered light from the sample based on their unique vibrational characteristics (fingerprints). The Raman scattering effect was predicted first theoretically by Smekal in 1923 and discovered by C.V. Raman in an experiment in 1928 [95], which is the basis of Raman spectroscopy. In the 1960s, a single-wavelength sources became possible owing to the advent of lasers, which promoted the experimental applications of Raman spectroscopy. Figure 4a illustrates the principle of elastic (Rayleigh) and inelastic scattering. When an incident light ray focuses on the sample through the microscope objective at a defined magnification, Rayleigh scattering, fluorescence, anti-Stokes and Stokes Raman scattering are produced [96]. Among them, fluorescence is harmful to Raman spectroscopy, and is usually suppressed. Anti-Stokes and Stokes Raman scattering is collected by a detector and their diagrammatic sketch is presented in Figure 4b. When an incident lightbeam focuses on the samples, photons interact with chemical bonds or electronic clouds and produce an oscillating polarization in the molecules, exciting the electrons to the virtual energy levels. As it is shown in Figure 4b, electrons on virtual energy levels transfer to the original energy level and emit photons due to the instability of electrons on virtual energy levels, which process is called Rayleigh scattering. There is no energy change in Rayleigh scattering. Rayleigh scattering does not carry related information about sample molecules, which is different from Raman scattering. Anti-Stokes and Stokes Raman scattering is inelastic scatting, in which the transition energy of electrons excited by incident photons are different from the energy of emitted photons of light. In Stokes Raman scattering, the frequency of the incident photons is higher than the emitted photons, meaning that the incident photons have a higher energy than emitted photons, while in anti-Stokes scattering, the incident photons are of lower energy than emitted photons. Besides, the intensity of anti-Stokes scattering is weaker than Stokes scattering, so Stokes scattering is more remarkable in experiments. Figure 4c shows a typical setup of the confocal micro-Raman spectroscopic system. The sample to be tested is placed on the sample stage, and the information of Raman scattering (mainly Stokes scattering) produced by the system is gathered by the final detector [97]. 

Through subsequent analysis using a computer system, the Raman spectrum with the appearance as shown in Figure 4e is obtained. Figure 4d illustrates the optical vibration modes of TMDs, which can be characterized by Raman spectroscopy. Different vibration modes store different molecules information, which can be reflected in Raman spectra. A_1g_ and E_2g_ are two typical vibration modes in TMDs. The positions of Raman shift of A_1g_ peak or E_2g_ peak are analyzed and used to clearly distinguish single and few layered TMDs as shown in Figure 4e.

### 3.2. Application of Raman Spectroscopy

Raman spectroscopy is a non-invasive and non-destructive technique, which requires almost no sample treatment, being suitable for solid state, aqueous conditions and gas phase, as well as film samples. Thus, the applications of Raman spectroscopy are greatly expanded. At present, Raman spectroscopy has a wide range of applications in the fields of chemistry, biomedicine, physics, and so on [100,101,102]. Raman spectroscopy is also made great contributions to the study of TE materials [103]. With the increasing technical requirements, Raman spectroscopy has now developed into multiple branches, e.g., resonance Raman spectroscopy, surface enhanced Raman spectroscopy [104,105], tip enhanced Raman spectroscopy [106], and micro Raman spectroscopy etc. Micro-Raman spectroscopy is one of the most important techniques to characterize nanomaterials, which can identify the number of layers of film materials [107], analyze the vibration modes of 2D materials in diverse case [71], and measure the thermal conductivity [22,108,109,110].

### 3.3. Raman Spectroscopy for Measuring Thermal Conductivity of 2D Materials

As one of the main parameters for characterizing TE performance, the thermal conductivity has been measured by employing Raman spectroscopy on some layered materials. The following sections discuss graphene, h-BN, BP, and TMDs in details.

#### 3.3.1. Graphene

Graphene is a single layer of carbon atoms bonded through *sp*^2^ hybridization with regular hexagonal honeycomb crystal structure exfoliated from graphite. The model diagram of graphene is shown in Figure 2. Graphene has attracted tremendous attention since its successful preparation, owing to its excellent optical [111], electrical [5], mechanical [112], and thermal properties [22,113]. Recently, graphene has also triggered great interest as a new type of TE material and led to a prosperous development [64,114,115,116]. However, its application in thermoelectrics is limited by the high thermal conductivity and the low Seebeck coefficient due to its gapless spectrum. Hence a series of methods for optimizing TE performance of graphene by enhancing its Seebeck coefficient or decreasing the thermal conductivity have emerged, including modification of graphene band structure, nanostructures [115,117], doping, and graphene-based polymer nanocomposites and so on [118,119]. That makes graphene a promising TE material and promotes its applications [120], such as TE cooling [121].

Raman spectroscopy as a versatile tool, provides a novel method in measuring the thermal conductivity and promotes the development of graphene on thermoelectricity in experiment. Before 2008, although a large number of theoretical studies showed that graphene has a high thermal conductivity [122,123], which has not been confirmed experimentally due to the lack of conventional methods for measuring 2D materials.

**Figure 5:**
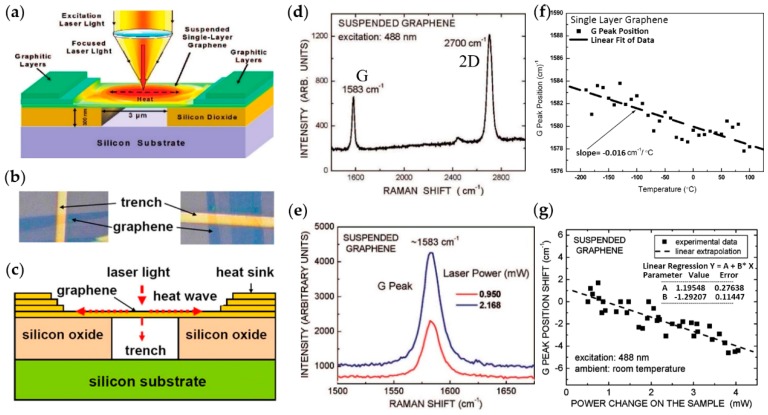
The group diagram for measuring the thermal conductivity of single graphene by micro-Raman spectroscopy. (**a**) Schematic of experiment model for measurement. In this schematic, the focused laser light exposure on a graphene layer suspended across a trench producing a hot spot and being incident light of Raman spectroscopy. The heat sink is graphitic layer to ensure good heat dissipation at the edge of the layer of graphene. Reproduced with permission from [22]. Copyright 2008, American Chemical Society. (**b**) The vertical scanning electron microscopy image of the suspended graphene flakes where one can clearly see the trench and suspended graphene. (**c**) The front view of experiment model. In this schematic, one can more clearly see that a hot spot generates heat waves inside single-layer graphene (SLG) propagating toward heat sinks. In addition, the silicon substrate and the upper silicon oxide are also given for clarity. Reproduced with permission from [125]. Copyright 2008, American Institute of Physics. (**d**) Raman spectrum of suspended graphene showing the G peak (at ~1583 cm^−1^) and 2D peak (at ~2700 cm^−1^) at room temperature excited at 488 nm. (**e**) Raman spectrum of G peak at two different laser power. The red and blue lines represent 0.950 and 2.168 mW, respectively. Weakly evolution of Raman shift of G peak occurs in different laser power. (**f**) Temperature dependence of the G peak frequency for the single layer graphene. The solid square black spots are experimental data of G peak position at a certain temperature and the fitted straight dash line slope is the temperature coefficient which is −0.016 cm^−1^/°C Reproduced with permission from [124]. Copyright 2007, American Chemical Society. (**g**) The G peak position shift dependence of total dissipated laser power excited at 488 nm. The fitted straight dash line slope is the power coefficient. The inserted chart illustrates a method of fitting data using linear regression equations where the values of parameter A and B are given. Reproduced with permission from [22]. Copyright 2008, American Chemical Society.

Balandin et al. developed noncontact micro-Raman spectroscopy to measure the thermal conductivity of graphene according to the temperature dependence of the frequency of G peak in the Raman spectra [22,124,125,126,127,128]. The schematic setup of the experimental device is shown in Figure 5a and details are shown in Figure 5b,c for clarity. The focused laser light exposes on the middle of suspended single-layer graphene (SLG), forming a hot spot and diffusing around by heat waves, which produces a stable temperature gradient on the graphene. At the same time, the laser is also acted as an incident source for Raman spectroscopy, and the Raman spectrum of SLG is gained by analyzing Raman scattering light, which is shown in Figure 5d. The position of G and 2D peaks are ~1583 cm^−1^ and ~2700 cm^−1^ at room temperature, respectively. When the laser power changes, the position of the peak will also change. Figure 5e is the Raman spectrum of G peak at two different laser powers and shows the weak evolution of Raman shift of G peak. For the graphene suspended on trench shown in Figure 5b, when the laser hot spot is much smaller than the width of the SLG sample, it can be considered that the heat waves move in two opposite directions toward the trench edges, assuming that temperature of the trench edges is consistent with the heat sink and room temperature. Then, the thermal conductivity of the sample can be expressed as:

(8) $$\kappa =\frac{d}{2A}\cdot \frac{∆P}{∆T}$$

where *d* is the distance from the middle of the suspended SLG to the heat sink with the temperature ambient *T* and cross-sectional area *A* = *h* × *W* (*h* and *W* is the thickness and width of SLG, respectively). Thus, the thermal conductivity of SLG will be calculated if the laser power absorbed ∆*P* by SLG and the resulting local temperature rise ∆*T* are measured out. The success of this work is benefited from a previous work on graphene by Calizo et al. This work demonstrated that the frequency of G peak in the Raman spectra of graphene on Si/SiO_2_ substrates is dependent on temperature, concluding the relation of:

*ω* = *ω*_0_ + *χ_T_T* (9)

where *ω* and *ω*_0_ are the frequencies of the G peak at temperature T and T extrapolated to 0 K, respectively. *χ_T_* is temperature coefficient, which defines the slope of the dependence [124]. In this work, *χ_T_* was calculated by the fitted straight dash line slope in Figure 5f. Through the processing of relation (8) and (9), the thermal conductivity can be expressed as:

(10) $$\kappa =\chi_{T}\cdot \frac{d}{2hW}\cdot {\left(\frac{\delta \omega }{\delta p}\right)}^{-1}$$

The above equation involves the changes of G peak frequency with laser power absorbed by SLG—*δω*/Δ*p*, which is the only unknown factor in the equation at present. Figure 5g is the G peak position shift dependence of total dissipated laser power, in which the fitted straight dash line slope is *δω*/*δP*_D_ (or *χ_P_*), where *P*_D_ is the total laser power, and *P*_D_ = *P* + *P*_Si_, where *P*_Si_ is laser power absorbed by Si. The amount of the power *P* absorbed by the suspended graphene can be evaluated through the calibration procedure with pyrolytic graphite (for detailed steps, see [22]). Thus, through the above process, the thermal conductivity about 5300 ± 480 W·m^−1^·K^−1^ of graphene at room temperature is obtained, which was the first time the challenge of measurement on thermal conductivity of graphene experimentally was overcome [22].

In addition, Chen et al. used micro-Raman spectroscopy to measure the thermal conductivity of monolayer graphene of variable sizes in vacuum and gaseous environments in 2011, which values range from (2600 ± 900) to (3100 ± 1000) W·m^−1^·K^−1^ near 350 K [129]. Lee et al. also measured the thermal conductivity in suspended pristine graphene by Raman spectroscopy in 2011, which values range from ~1800 W·m^−1^·K^−1^ near 325 K to ∼710 W·m^−1^·K^−1^ at 500 K [130]. These successes of experiments on measuring thermal conductivity by Raman spectroscopy pave a new way to measure TE performance of 2D TE materials, and are vital to the advancement of 2D thermoelectricity.

#### 3.3.2. h-BN

Boron nitride (BN), which exists in Nature, consists of equal numbers of boron (B) and nitrogen (N) atoms [131]. h-BN is one form of the BN, which is the most stable BN phase under ambient conditions [132]. An atomic model of h-BN is shown in Figure 2. As it is shown, h-BN is one kind of 2D materials, which has a layered crystal structure with alternating B and N atoms. It is also called “white graphene” as a single-layer h-BN nanosheet can be regarded as a graphene analogue [7]. Similar structures often lead to some common features, such as high mechanical strength and thermal conductivity, as well as good lubrication [132]. There are also many properties distinct from graphene in h-BN, and it is significant to explore these dissimilarities and employ them in what is not applied in graphene. The most striking difference is that h-BN possesses a huge bandgap while graphene is gapless, so that it can act as an insulating layer or dielectric substrate [133]. h-BN was rarely investigated separately as TE materials due to its intrinsic properties about the low electrical conductivity and the high thermal conductivity.

**Figure 6:**
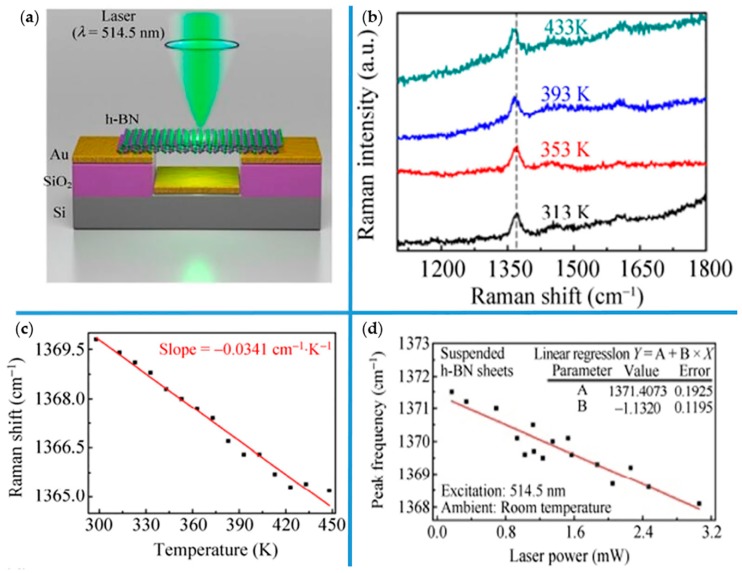
The group diagram for measuring the thermal conductivity of single layered h-BN sheet by micro-Raman spectroscopy. (**a**) Schematic of experiment model for measuring the thermal conductivity of h-BN. (**b**) Raman spectrum of single layer h-BN at different temperature where Raman frequency evolution of Raman E_2g_ mode with a variety of temperature is clearly seen. (**c**) and (**d**) present temperature- and laser power-dependent peak frequency of E_2g_ mode in suspended h-BN sheets, respectively. The explanation of the fitted lines and inserted chart are similar to Figure 5. Reproduced with permission from [108]. Copyright 2014, Springer International Publishing.

The thermal conductivity of atomically thin h-BN has been explored [108,110,134,135,136,137,138], experimentally. It can reach to 484 W·m^−1^·K^−1^ in bilayer h-BN as measured by suspended prepatterned microstructures [137], and around 360 W·m^−1^·K^−1^ in 11-layer h-BN measured by a microbridge device with built-in thermometers [138] at room temperature. Zhou et al. reported an experiment that used noncontact micro-Raman spectroscopy as another excellent method to measure thermal conductivity of layered h-BN in 2014 [108]. Raman spectroscopy plays an important role in evaluating the thermal conductivity of thin h-BN sheets. Figure 6a shows the schematic of an experimental setup for measuring the thermal conductivity of h-BN sheets by micro-Raman spectroscopy. A 514.5 nm laser excited with a ~1 μm spot size is focused on the middle of suspended layered h-BN under ambient conditions, depending on golden heat sink to dissipate heat and maintain a stable temperature gradient. For evaluating the thermal conductivity, there are two parameters that should be measured by this setup according to Equation (10). The first is the first-order temperature coefficient *χ_T_* in the Raman scattering. On the basis of the results described in [108], the Raman E_2g_ mode red-shifts of layered h-BN are sensitive to surrounding temperature, in that Raman E_2g_ mode peak shifts 4.0 cm^–1^ with the temperature increasing from 313 to 433 K as it shown in Figure 6b. The temperature-dependent peak frequency of E_2g_ mode of single-layer h-BN is shown in Figure 6c, and from the fitted line, first-order temperature coefficient *χ_T_* is obtained. The other parameter for the thermal conductivity measurement of h-BN is the dependence of the Raman E_2g_ mode frequency on the laser power *δω*/*δP* (*χ_P_*). Figure 6d is the image of E_2g_ mode peak frequency as a function of the laser power, and the *δω*/*δP* can be extracted from the slope of fitted line. Then, based on acquiring the ratio of laser power absorbed by h-BN, the thermal conductivity can be calculated in the range from 227 to 280 W·m^−1^·K^−1^ at room temperature.

#### 3.3.3. BP

**Figure 7:**
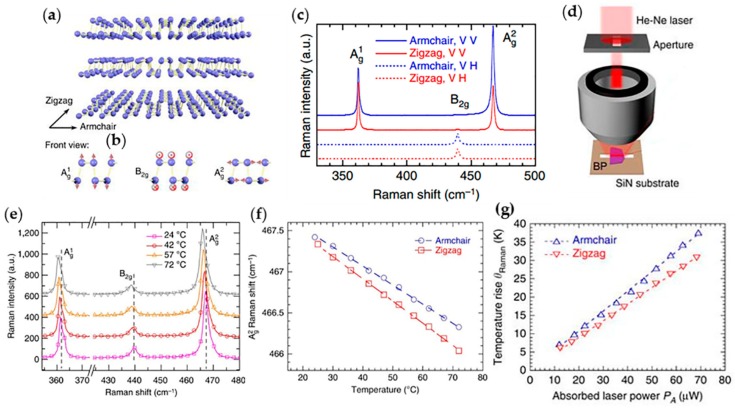
Structure characterization of BP and the process on its thermal conductivity measurements using micro-Raman technique. (**a**) Diagram of the lattice structure of BP. Directions of zigzag and armchair are indicated. (**b**) Atomic vibrational patterns of A_g_^1^, B_2g_ and A_g_^2^ phonon modes from the front view. (**c**) Raman spectra of BP at different directions and vibrational patterns. ‘VV’ and ‘VH’ are two configurations standing for different directions of laser polarization to gain the A_g_ and B_2g_ vibrational patterns. Zigzag is represented by red lines and armchair is represented by blue lines. (**d**) Illustration of experiment model for measuring suspended BP flakes. Here He-Ne laser through aperture and collecting mirror exposures on the suspended BP laying SiN substrate. (**e**) The Raman spectra of BP flakes at 72, 57, 42 and 24 °C. The three modes of vibration patterns are marked with dashed lines in the diagram so that the Raman shift of them are clearly visible. (**f**) The A_g_^2^ Raman shift as a function of temperature under armchair- and zigzag-polarized laser. The fitted straight dash line slopes are the temperature coefficients. (**g**) The temperature rise as a function of absorbed laser power of BP film along armchair and zigzag directions. Reproduced with permission from [109]. Copyright 2015, Nature Publishing Group.

BP is an emerging 2D material with potential applications in electronics and optoelectronics [139,140,141,142,143,144,145]. BP is a layered material as shown in Figure 2, in which individual layers stack together through the van der Waals force, much like bulk graphite, and was also successfully prepared by mechanical exfoliation. Monolayer BP is composed of a puckered honeycomb structure, in which one P atom bonds with the other three. The structure of BP is anisotropic with the zigzag and the armchair directions marked in Figure 7a, which results in its anisotropic transport properties [7,136,146,147,148]. Importantly, the bandgap of BP is tunable depending on its thickness, and is highly sensitive to the plane strain and edge structures on prediction [149,150]. Both the intrinsic in-plane anisotropy and the moderate bandgap of BP also promote considerable development of TE application [151,152,153,154,155,156,157]. In addition, BP is theoretically predicted to be a promising TE material [154,155,158,159,160] due to its high carrier mobility [161], large Seebeck coefficient, as well as relatively low thermal conductivity [109,162], and the experimental research on thermoelectricity of BP is also in full swing [163].

However, it is apparent that the experimental research is much less than the theoretical prediction on thermoelectricity of BP, because experimental measurements are much more complex and difficult. Raman spectroscopy is a very powerful tool to characterize flakes, and using this technique, previous studies reported the identification of thicknesses for BP [164]. The present status of Raman spectroscopy in BP are concluded by Ribeiro et al. [165]. More importantly, Raman spectroscopy is also a perfect method in measuring thermal conductivity of layered BP [109,162,166], which will lead to a prosperous advancement in thermoelectricity of BP.

The measurement of suspended layered 2D materials by micro-Raman spectroscopy was presented in graphene and h-BN above, whereas BP possesses anisotropic properties and need to distinguish the zigzag and armchair axes using polarized Raman spectroscopy firstly, which was also used by Wu et al. [167] and Ribeiro et al. [168] in 2015 on BP. Figure 7b is the diagram of Raman vibration modes of BP, including in-plane A_g_^1^ and A_g_^2^ mode, as well as the out-of-plane B_2g_ mode in front view. The different directions show different intensity ratios of A_g_^2^ to A_g_^1^, where the A_g_^2^/A_g_^1^ becomes larger (~2) with armchair-polarized laser excitation and smaller (~1) with zigzag-polarized laser excitation as shown in Figure 7c, which serves as Raman signatures of armchair and zigzag lattice axes and is a basis of studying the anisotropic thermal conductivity of BP. Figure 7d is the illustration of the experimental setup of micro-Raman spectroscopy. By collecting and analyzing the Raman scattering light of the sample, the changes of Raman shift with laser power can be figured out. In addition, the Raman spectra of BP at different temperature are shown in Figure 7e, from which we can fit straight lines of the Ag^2^ mode in zigzag and armchair axes as shown in Figure 7f. A series of Raman spectra can be obtained under various incident laser power settings, which give the laser power coefficient. In addition to the previously obtained temperature coefficient, the laser-power-dependent temperature rise of BP film is acquired as shown in Figure 7g, which can be expressed as *δT*/*δP_A_*. Then, through auxiliary calculations, the thermal conductivity of layered BP is evaluated eventually, which is about 10–20 W·m^−1^·K^−1^ for thin-film BP [109].

#### 3.3.4. TMDs

TMDs, which are semiconductors of the type MX_2_ (M = W, Mo; X = S, Se, Te), are a burgeoning class of 2D materials with novel physical properties and are expected to become an alternative to graphene. TMDs are also layered materials with van der Waals interactions between layers, and a diagram of their structure is shown in Figure 2. Note that TMDs exist in several structural phases owing to different coordination spheres of the transition metal atoms, including trigonal prismatic (2H), distorted octahedral (1T) and dimerized (1T′) phases. TMDs show excellent properties in electronics, optoelectronics, mechanics, chemistry and thermoelectricity, which will enable TMDs to have great potential in the future of technology [169,170,171,172,173,174,175]. It is interesting to understand the excellent TE properties of TMDs, which will promote the advancement of thermoelectricity. In recent years, there have been lots of theoretical studies on the TE properties of TMDs by first-principles and density functional calculations, as well as semiclassical Boltzmann transport theory, predicting that TMDs have a competitive TE performance, due to their adjustable electronic properties and high electrical conductivity confined in their 2D planes [16,66,69,70,175,176,177,178]. The low thermal conductivity of TMDs is also a critical factor resulting in excellent TE performance, which is from 0.05 to 62.2 W·m^−1^·K^−1^ in different experiments reviewed by Wang et al. in 2017 [136]. In these experiments, Raman spectroscopy acts a pivotal part in the measurement, especially in MoS_2_ and WS_2_. The following emphasizes on the measurement of MoS_2_ by Raman spectroscopy.

**Figure 8:**
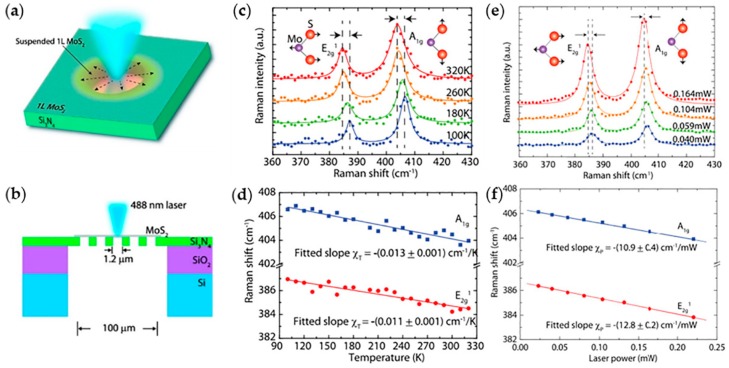
Thermal conductivity measurements of MoS_2_ using micro-Raman technique. (**a**) Illustration of the experimental setup for measuring. (**b**) The sectional view of (**a**). Here suspended monolayer MoS_2_ over the holes in the 20 nm thick Si_3_N_4_ on SiO_2_/Si substrate is presented. (**c**) The Raman spectra of suspended monolayer MoS_2_ at 320, 260, 180 and 100 K. The atomic vibrational modes of in-plane E_2g_^1^ and out-of-plane A_1g_ are inserted in the diagram for clarity. (**d**) Raman peak frequencies of both Raman A_1g_ and E_2g_^1^ modes as a function of temperature. The fitted slope resulting linear temperature coefficients are shown. The blue line is the A_1g_ mode and red line is the E_2g_^1^ mode. (**e**) The Raman spectra of suspended monolayer MoS_2_ at laser power of 0.164, 0.104, 0.059 and 0.040 mW, respectively. The atomic vibrational modes of A_1g_ and E_2g_^1^ are inserted in the diagram for clarity. (**f**) Raman peak frequencies of both Raman A_1g_ and E_2g_^1^ modes as a function of temperature. The fitted slope resulting linear power coefficients are shown. Reproduced with permission from [110]. Copyright 2014, American Chemical Society.

MoS_2_ is one of the most stable layered TMDs, and it has achieved remarkable results in terms of electronic, optoelectronic devices [169,179,180], as well as thermoelectricity [88,181]. Previous works provided a detailed process on measuring thermal conductivity of suspended layered MoS_2_ by micro-Raman spectroscopy [110,182,183,184]. Figure 8a is the schematic of the experiment setup and Figure 8b is the sectional view of 8(a) for clarity. In this experiment, due to the low thermal conductivity of MoS_2_, there is a porous Si_3_N_4_ plate with the holes 1.2 μm suspended on SiO_2_/Si substrate, and the MoS_2_ sheet was placed thereon, as shown in Figure 8b. At different temperature, the atomic vibrational modes of MoS_2_ changes, which contribute to Raman spectra offset as shown in Figure 8c. According to the offset, Figure 8d gives the temperature dependence of Raman peak frequencies from 100 to 320 K for the A_1g_ and E_2g_^1^ modes. By the fitted slopes, the temperature coefficient can be calculated. Then, from the offset of Raman spectra of the two modes at different laser power shown in Figure 8e, the Raman peak frequencies as a function of laser power are obtained, which show in in Figure 8f. By the fitted slopes, the laser power coefficient can be calculated. Finally, the resulting thermal conductivity *κ* = (34.5 ± 4) W·m^−1^·K^−1^ of MoS_2_ at room temperature is evaluated on the basis of the temperature coefficient and laser power coefficient.

The Table 1 summarizes the above data.

From the table, one can clearly see that the thermal conductivities of graphene and h-BN are much more than those of BP and MoS_2_. Therefore, the laser power is lower in the experiment of BN and MoS_2_ to avoid local overheating and sample damage. The magnitude of the temperature coefficient is same, which may depend on the similarity of the mechanism of temperature influencing the phonon vibration modes. By analyzing the processes of measuring thermal conductivities of graphene, h-BN, BP, and MoS_2_ employing Raman spectroscopy, a promising method in evaluating the thermal conductivity of 2D materials is obtained, which paves the way for efficient thermal management in 2D layered materials.

## 4. Conclusions and Outlook

In summary, 2D TE materials, which have been explored and studied widely in the past few years, will become a promising direction on optimizing TE performance. Similar to bulk materials, the measurement on TE performance of 2D materials also involves the electrical and thermal conductivities, as well as the Seebeck coefficient of specimen. Raman Spectroscopy is a precise and powerful method in materials characterization, which is known as a spectroscopic ‘Swiss-Army Knife’. The measurement of the thermal conductivity of 2D materials has been achieved by Raman spectroscopy. Based on it, making an electric test on the specimen in the Raman resting environment at a controllable temperature, obtaining the ∆*V*/∆*T*, and ∆*V*/∆*I*, which contributes to calculate the Seebeck coefficient and electrical conductivity, respectively. Eventually, the *ZT* of 2D TE materials will be evaluated by Equation (3). It is worth mentioning that, measuring *ZT* by Raman Spectroscopy combined thermal properties with electrical properties in bulk materials was achieved by Chen et al. and Yu et al. in recent years [185,186], which boosts the advancement of TE research. Extending it to the field of 2D materials will further promote the development of thermoelectricity.

Nevertheless, some challenges still remain in the 2D TE materials and the measurement of their performances:

(1) Advancement of 2D TE materials. Studies on 2D TE materials are still in their infancy, so the actual TE performances in the experiments or devices are not ideal enough, and the practical applications are still limited. It is necessary to continue to develop 2D TE materials and promote their performance. To note, high pressure applied to materials can strikingly improve TE performance [185,186,187], which provides a meaningful idea for advancement of 2D TE materials.
(2) The measurement of electric properties of 2D materials. It is difficult to accurately measure the electric properties of 2D materials due to the miniaturization of the scales.

The last few years have witnessed the exciting development of TE materials, in which Raman spectroscopy has undoubtedly made a great contribution, especially for 2D TE materials. With unremitting efforts and struggles of researchers, Raman spectroscopy will boost further advancements of TE materials, contributing to resolving the contradiction between energy waste and insufficient resources in the future.

## Figures and Tables

**Table 1:** Comparison of the thermal conductivity measurement parameters of different layered samples by Raman spectroscopy at room temperature.

Sample	Trench Width (Hole Diameter)	*χ_T_* (cm^−1^/K)	*χ_P_* (cm^−1^/mW)	κ (W m^−1^ K^−1^)	Ref.
Graphene	3 μm	−0.0162 ± 0.0020	−1.29 ± 0.11	5300 ± 480	Balandin et al. [22], Calizo et al. [124]
h-BN	7 μm	−0.0341 ± 0.0012	−1.13 ± 0.12	227~280	Zhou et al. [108]
BP	- *	Armchair −0.019~0.024 Zigzag −0.022~0.028	- *	10~20	Luo et al. [109]
MoS_2_	1.2 μm	A_1g_ −0.013 ± 0.001 E_2g_^1^ −0.011 ± 0.001	A_1g_ −10.9 ± 0.4 E_2g_^1^ −12.8 ± 0.2	34.5 ± 4	Yan et al. [110]

* The trench width and *χ_P_* of BP are not provided in Ref. [109].

## References

[1] Snyder G.J., Toberer E.S. (2008). Complex thermoelectric materials. Nat. Mater..

[2] Bell L.E. (2008). Cooling, Heating, Generating Power, and Recovering Waste Heat with Thermoelectric Systems. Science.

[3] Tritt T.M., Subramanian M.A. (2011). Thermoelectric Materials, Phenomena, and Applications: A Bird’s Eye View. MRS Bull..

[4] Gorai P., Stevanović V., Toberer E.S. (2017). Computationally guided discovery of thermoelectric materials. Nat. Rev. Mater..

[5] Novoselov K.S., Geim A.K., Morozov S.V., Jiang D., Zhang Y., Dubonos S.V., Grigorieva I.V., Firsov A.A. (2004). Electric Field Effect in Atomically Thin Carbon Films. Science.

[6] Xia F., Wang H., Xiao D., Dubey M., Ramasubramaniam A. (2014). Two-dimensional material nanophotonics. Nat. Photonics.

[7] Tan C., Cao X., Wu X.-J., He Q., Yang J., Zhang X., Chen J., Zhao W., Han S., Nam G.-H. (2017). Recent Advances in Ultrathin Two-Dimensional Nanomaterials. Chem. Rev..

[8] Xu M., Liang T., Shi M., Chen H. (2013). Graphene-like two-dimensional materials. Chem. Rev..

[9] Wang C., Wu X., Ma Y., Mu G., Li Y., Luo C., Xu H., Zhang Y., Yang J., Tang X. (2018). Metallic few-layered VSe_2_ nanosheets: High two-dimensional conductivity for flexible in-plane solid-state supercapacitors. J. Mater. Chem..

[10] Luo C., Wang C., Wu X., Zhang J., Chu J. (2017). In Situ Transmission Electron Microscopy Characterization and Manipulation of Two-Dimensional Layered Materials beyond Graphene. Small.

[11] Liu S., Wu X., Zhang D., Guo C., Wang P., Hu W., Li X., Zhou X., Xu H., Luo C. (2017). Ultrafast Dynamic Pressure Sensors Based on Graphene Hybrid Structure. ACS Appl. Mat. Interfaces.

[12] Cao Z.-Y., Hu J.-W., Goncharov A.F., Chen X.-J. (2018). Nontrivial metallic state of MoS_2_. Phys. Rev. B.

[13] Sharma S., Singh N., Schwingenschlögl U. (2018). Two-Dimensional Tellurene as Excellent Thermoelectric Material. ACS Appl. Energy Mater..

[14] Hung N.T., Nugraha A.R.T., Saito R. (2017). Two-dimensional InSe as a potential thermoelectric material. Appl. Phys. Lett..

[15] Khazaei M., Arai M., Sasaki T., Estili M., Sakka Y. (2014). Two-dimensional molybdenum carbides: Potential thermoelectric materials of the MXene family. Phys. Chem. Chem. Phys..

[16] Choudhary N., Islam M.A., Kim J.H., Ko T.-J., Schropp A., Hurtado L., Weitzman D., Zhai L., Jung Y. (2018). Two-dimensional transition metal dichalcogenide hybrid materials for energy applications. Nano Today.

[17] Wan C., Tian R., Kondou M., Yang R., Zong P., Koumoto K. (2017). Ultrahigh thermoelectric power factor in flexible hybrid inorganic-organic superlattice. Nat. Commun..

[18] Bulman G., Barletta P., Lewis J., Baldasaro N., Manno M., Bar-Cohen A., Yang B. (2016). Superlattice-based thin-film thermoelectric modules with high cooling fluxes. Nat. Commun..

[19] Lee M.J., Ahn J.H., Sung J.H., Heo H., Jeon S.G., Lee W., Song J.Y., Hong K.H., Choi B., Lee S.H. (2016). Thermoelectric materials by using two-dimensional materials with negative correlation between electrical and thermal conductivity. Nat. Commun..

[20] Hippalgaonkar K., Wang Y., Ye Y., Qiu D.Y., Zhu H., Wang Y., Moore J., Louie S.G., Zhang X. (2017). High thermoelectric power factor in two-dimensional crystals of MoS_2_. Phys. Rev. B.

[21] Zhang S., Zhang N., Zhao Y., Cheng T., Li X., Feng R., Xu H., Liu Z., Zhang J., Tong L. (2018). Spotting the differences in two-dimensional materials - the Raman scattering perspective. Chem. Soc. Rev..

[22] Balandin A.A., Ghosh S., Bao W., Calizo I., Teweldebrhan D., Miao F., Lau C.N. (2008). Superior Thermal Conductivity of Single-Layer Graphene. Nano Lett..

[23] Leonov V., Vullers R.J.M. (2009). Wearable Thermoelectric Generators for Body-Powered Devices. J. Electron. Mater..

[24] Suarez F., Parekh D.P., Ladd C., Vashaee D., Dickey M.D., Öztürk M.C. (2017). Flexible thermoelectric generator using bulk legs and liquid metal interconnects for wearable electronics. Appl. Energy.

[25] Baranowski L.L., Snyder G.J., Toberer E.S. (2012). Concentrated solar thermoelectric generators. Energy Environ. Sci..

[26] Kristiansen N.R., Snyder G.J., Nielsen H.K., Rosendahl L. (2012). Waste Heat Recovery from a Marine Waste IncineratorUsing a Thermoelectric Generator. J. Electron. Mater..

[27] He W., Zhang G., Zhang X., Ji J., Li G., Zhao X. (2015). Recent development and application of thermoelectric generator and cooler. Appl. Energy.

[28] Chen L.-C., Jiang B.-B., Yu H., Pang H.-J., Su L., Shi X., Chen L.-D., Chen X.-J. (2018). Thermoelectric properties of polycrystalline palladium sulfide. RSC Adv..

[29] Pang H.-J., Fu C.-G., Yu H., Chen L.-C., Zhu T.-J., Chen X.-J. (2018). Origin of efficient thermoelectric performance in half-Heusler FeNb_0.8_Ti_0.2_Sb. J. Appl. Phys..

[30] Wang X., Wang H., Liu B. (2018). Carbon Nanotube-Based Organic Thermoelectric Materials for Energy Harvesting. Polymers.

[31] Xie W., Tang X., Yan Y., Zhang Q., Tritt T.M. (2009). Unique nanostructures and enhanced thermoelectric performance of melt-spun BiSbTe alloys. Appl. Phys. Lett..

[32] Chung D.-Y., Hogan T., Brazis P., Rocci-Lane M., Kannewurf C., Bastea M., Uher C., Kanatzidis M.G. (2000). CsBi_4_Te_6_: A High-Performance Thermoelectric Material for Low-Temperature Applications. Science.

[33] Poudel B., Hao Q., Ma Y., Lan Y., Minnich A., Yu B., Yan X., Wang D., Muto A., Vashaee D. (2008). High-Thermoelectric Performance of Nanostructured Bismuth Antimony Telluride Bulk Alloys. Science.

[34] Shanyu W., Han L., Ruiming L., Gang Z., Xinfeng T. (2013). Metal nanoparticle decorated N-type Bi_2_Te_3_ -based materials with enhanced thermoelectric performances. Nanotechnology.

[35] Joshi G., Lee H., Lan Y., Wang X., Zhu G., Wang D., Gould R.W., Cuff D.C., Tang M.Y., Dresselhaus M.S. (2008). Enhanced Thermoelectric Figure-of-Merit in Nanostructured P-type Silicon Germanium Bulk Alloys. Nano Lett..

[36] Minnich A.J., Lee H., Wang X.W., Joshi G., Dresselhaus M.S., Ren Z.F., Chen G., Vashaee D. (2009). Modeling study of thermoelectric SiGe nanocomposites. Phys. Rev. B.

[37] Heremans J.P., Jovovic V., Toberer E.S., Saramat A., Kurosaki K., Charoenphakdee A., Yamanaka S., Snyder G.J. (2008). Enhancement of Thermoelectric Efficiency in PbTe by Distortion of the Electronic Density of States. Science.

[38] Pei Y., Lensch-Falk J., Toberer E.S., Medlin D.L., Snyder G.J. (2011). High Thermoelectric Performance in PbTe Due to Large Nanoscale Ag_2_Te Precipitates and La Doping. Adv. Funct. Mater..

[39] Guo J.Q., Geng H.Y., Ochi T., Suzuki S., Kikuchi M., Yamaguchi Y., Ito S. (2012). Development of Skutterudite Thermoelectric Materials and Modules. J. Electron. Mater..

[40] Elsheikh M.H., Sabri M.F.M., Said S.M., Miyazaki Y., Masjuki H.H., Shnawah D.A., Naito S., Bashir M.B.A. (2017). Rapid preparation of bulk Al_x_Yb_0.25_Co_4_Sb_12_ (x = 0, 0.1, 0.2, 0.3) skutterudite thermoelectric materials with high figure of merit ZT = 1.36. J. Mater. Sci..

[41] Zeier W.G., Schmitt J., Hautier G., Aydemir U., Gibbs Z.M., Felser C., Snyder G.J. (2016). Engineering half-Heusler thermoelectric materials using Zintl chemistry. Nat. Rev. Mater..

[42] Wang J., Lebedev O.I., Lee K., Dolyniuk J.A., Klavins P., Bux S., Kovnir K. (2017). High-efficiency thermoelectric Ba_8_Cu_14_Ge_6_P_26_: Bridging the gap between tetrel-based and tetrel-free clathrates. Chem. Sci..

[43] Zhao L.-D., Tan G., Hao S., He J., Pei Y., Chi H., Wang H., Gong S., Xu H., Dravid V.P. (2016). Ultrahigh power factor and thermoelectric performance in hole-doped single-crystal SnSe. Science.

[44] Zhao L.-D., Lo S.-H., Zhang Y., Sun H., Tan G., Uher C., Wolverton C., Dravid V.P., Kanatzidis M.G. (2014). Ultralow thermal conductivity and high thermoelectric figure of merit in SnSe crystals. Nature.

[45] Wei P.-C., Bhattacharya S., He J., Neeleshwar S., Podila R., Chen Y.Y., Rao A.M. (2016). The intrinsic thermal conductivity of SnSe. Nature.

[46] He J., Tritt T.M. (2017). Advances in thermoelectric materials research: Looking back and moving forward. Science.

[47] Champier D. (2017). Thermoelectric generators: A review of applications. Energy Convers. Manag..

[48] Dresselhaus M.S., Dresselhaus G., Sun X., Zhang Z., Cronin S.B., Koga T. (1999). Low-dimensional thermoelectric materials. Phys. Solid State.

[49] Dresselhaus M.S., Chen G., Tang M.Y., Yang R.G., Lee H., Wang D.Z., Ren Z.F., Fleurial J.P., Gogna P. (2007). New Directions for Low-Dimensional Thermoelectric Materials. Adv. Mater..

[50] Hicks L.D., Dresselhaus M.S. (1993). Thermoelectric figure of merit of a one-dimensional conductor. Phys. Rev. B.

[51] Hicks L.D., Dresselhaus M.S. (1993). Effect of quantum-well structures on the thermoelectric figure of merit. Phys. Rev. B.

[52] Li J.-F., Liu W.-S., Zhao L.-D., Zhou M. (2010). High-performance nanostructured thermoelectric materials. NPG Asia Mater..

[53] Kanatzidis M.G. (2010). Nanostructured Thermoelectrics: The New Paradigm?. Chem. Mater..

[54] Hochbaum A.I., Chen R., Delgado R.D., Liang W., Garnett E.C., Najarian M., Majumdar A., Yang P. (2008). Enhanced thermoelectric performance of rough silicon nanowires. Nature.

[55] Ma Y., Hao Q., Poudel B., Lan Y., Yu B., Wang D., Chen G., Ren Z. (2008). Enhanced Thermoelectric Figure-of-Merit in P-type Nanostructured Bismuth Antimony Tellurium Alloys Made from Elemental Chunks. Nano Lett..

[56] Lan Y., Minnich A.J., Chen G., Ren Z. (2010). Enhancement of Thermoelectric Figure-of-Merit by a Bulk Nanostructuring Approach. Adv. Funct. Mater..

[57] Wu X., Luo C., Hao P., Sun T., Wang R., Wang C., Hu Z., Li Y., Zhang J., Bersuker G. (2018). Probing and Manipulating the Interfacial Defects of InGaAs Dual-Layer Metal Oxides at the Atomic Scale. Adv. Mater..

[58] Wu X., Yu K., Cha D., Bosman M., Raghavan N., Zhang X., Li K., Liu Q., Sun L., Pey K. (2018). Atomic Scale Modulation of Self-Rectifying Resistive Switching by Interfacial Defects. Adv. Sci..

[59] Zhu Y.B., Zheng K., Wu X., Ang L.K. (2017). Enhanced stability of filament-type resistive switching by interface engineering. Sci. Rep..

[60] Lanza M., Wong H.S.P., Pop E., Ielmini D., Strukov D., Regan B.C., Larcher L., Villena M.A., Yang J.J., Goux L. (2018). Recommended Methods to Study Resistive Switching Devices. Adv. Electron. Mater..

[61] Wu X., Mei S., Bosman M., Raghavan N., Zhang X., Cha D., Li K., Pey K.L. (2015). Evolution of Filament Formation in Ni/HfO_2_/SiOx/Si-Based RRAM Devices. Adv. Electron. Mater..

[62] Wu X., Pey K.L., Zhang G., Bai P., Li X., Liu W.H., Raghavan N. (2010). Electrode material dependent breakdown and recovery in advanced high-κ gate stacks. Appl. Phys. Lett..

[63] Liang F., Xu H., Wu X., Wang C., Luo C., Zhang J. (2018). Raman spectroscopy characterization of two-dimensional materials. Chin. Phys. B.

[64] Xu Y., Li Z., Duan W. (2014). Thermal and thermoelectric properties of graphene. Small.

[65] Xiao H., Cao W., Ouyang T., Xu X., Ding Y., Zhong J. (2018). Thermoelectric properties of graphene nanoribbons with surface roughness. Appl. Phys. Lett..

[66] Wickramaratne D., Zahid F., Lake R.K. (2014). Electronic and thermoelectric properties of few-layer transition metal dichalcogenides. J. Chem. Phys..

[67] Chen K.-X., Wang X.-M., Mo D.-C., Lyu S.-S. (2015). Thermoelectric Properties of Transition Metal Dichalcogenides: From Monolayers to Nanotubes. J. Phys. Chem. C.

[68] Kumar S., Schwingenschlögl U. (2015). Thermoelectric Response of Bulk and Monolayer MoSe_2_ and WSe_2_. Chem. Mater..

[69] Huang W., Da H., Liang G. (2013). Thermoelectric performance of MX_2_ (M = Mo,W; X = S,Se) monolayers. J. Appl. Phys..

[70] Zhang G., Zhang Y.-W. (2017). Thermoelectric properties of two-dimensional transition metal dichalcogenides. J. Mater. Chem. C.

[71] Kim H., Anasori B., Gogotsi Y., Alshareef H.N. (2017). Thermoelectric Properties of Two-Dimensional Molybdenum-Based MXenes. Chem. Mater..

[72] Gandi A.N., Alshareef H.N., Schwingenschlögl U. (2016). Thermoelectric Performance of the MXenes M_2_CO_2_ (M = Ti, Zr, or Hf). Chem. Mater..

[73] Gao Y., Shi W., Wang W., Wang Y., Zhao Y., Lei Z., Miao R. (2014). Ultrasonic-Assisted Production of Graphene with High Yield in Supercritical CO_2_ and Its High Electrical Conductivity Film. Ind. Eng. Chem. Res..

[74] Radisavljevic B., Radenovic A., Brivio J., Giacometti V., Kis A. (2011). Single-layer MoS_2_ transistors. Nat. Nanotechnol..

[75] Demirci B.U., Miele P., Yot G.P. (2015). Boron-Based (Nano-)Materials: Fundamentals and Applications. Crystals.

[76] Anasori B., Xie Y., Beidaghi M., Lu J., Hosler B.C., Hultman L., Kent P.R.C., Gogotsi Y., Barsoum M.W. (2015). Two-Dimensional, Ordered, Double Transition Metals Carbides (MXenes). ACS Nano.

[77] Churchill H.O.H., Jarillo-Herrero P. (2014). Phosphorus joins the family. Nat. Nanotechnol..

[78] Novoselov K.S., Mishchenko A., Carvalho A., Castro Neto A.H. (2016). 2D materials and van der Waals heterostructures. Science.

[79] Venkatasubramanian R., Siivola E., Colpitts T., O’Quinn B. (2001). Thin-film thermoelectric devices with high room-temperature figures of merit. Nature.

[80] Harman T.C., Taylor P.J., Walsh M.P., LaForge B.E. (2002). Quantum Dot Superlattice Thermoelectric Materials and Devices. Science.

[81] Tian R., Wan C., Wang Y., Wei Q., Ishida T., Yamamoto A., Tsuruta A., Shin W., Li S., Koumoto K. (2017). A solution-processed TiS_2_/organic hybrid superlattice film towards flexible thermoelectric devices. J. Mater. Chem..

[82] Park N.-W., Ahn J.-Y., Cho N.-K., Park J.-S., Umar A., Lee S.-K. (2017). All In-Plane Thermoelectric Properties of Atomic Layer Deposition-Grown Al_2_O_3_/ZnO Superlattice Film in the Temperature Range from 300 to 500 K. Sci. Adv. Mater..

[83] He Y., Léonard F., Medlin D.L., Baldasaro N., Temple D.S., Barletta P., Spataru C.D. (2018). High-Efficiency Thin-Film Superlattice Thermoelectric Cooler Modules Enabled by Low Resistivity Contacts. Adv. Electron. Mater..

[84] Qin D., Yan P., Ding G., Ge X., Song H., Gao G. (2018). Monolayer PdSe_2_: A promising two-dimensional thermoelectric material. Sci. Rep..

[85] Naghavi S.S., He J., Xia Y., Wolverton C. (2018). Pd_2_Se_3_ Monolayer: A Promising Two-Dimensional Thermoelectric Material with Ultralow Lattice Thermal Conductivity and High Power Factor. Chem. Mater..

[86] Zhao T., Sun Y., Shuai Z., Wang D. (2017). GeAs_2_: A IV–V Group Two-Dimensional Semiconductor with Ultralow Thermal Conductivity and High Thermoelectric Efficiency. Chem. Mater..

[87] Tayari V., Senkovskiy B.V., Rybkovskiy D., Ehlen N., Fedorov A., Chen C.Y., Avila J., Asensio M., Perucchi A., di Pietro P. (2018). Quasi-two-dimensional thermoelectricity in SnSe. Phys. Rev. B.

[88] Huang W., Luo X., Gan C.K., Quek S.Y., Liang G. (2014). Theoretical study of thermoelectric properties of few-layer MoS_2_ and WSe_2_. Phys. Chem. Chem. Phys..

[89] Li B., Huang L., Zhao G., Wei Z., Dong H., Hu W., Wang L.-W., Li J. (2016). Large-Size 2D β-Cu_2_S Nanosheets with Giant Phase Transition Temperature Lowering (120 K) Synthesized by a Novel Method of Super-Cooling Chemical-Vapor-Deposition. Adv. Mater..

[90] Chi H., Kim H., Thomas J.C., Shi G., Sun K., Abeykoon M., Bozin E.S., Shi X., Li Q., Shi X. (2014). Low-temperature structural and transport anomalies in Cu_2_Se. Phys. Rev. B.

[91] Zhao L.-D., He J., Berardan D., Lin Y., Li J.-F., Nan C.-W., Dragoe N. (2014). BiCuSeO oxyselenides: New promising thermoelectric materials. Energy Environ. Sci..

[92] Wang C., Chen F., Sun K., Chen R., Li M., Zhou X., Sun Y., Chen D., Wang G. (2018). Contributed Review: Instruments for measuring Seebeck coefficient of thin film thermoelectric materials: A mini-review. Rev. Sci. Instrum..

[93] Jiang P., Qian X., Gu X., Yang R. (2017). Probing Anisotropic Thermal Conductivity of Transition Metal Dichalcogenides MX_2_ (M = Mo, W and X = S, Se) using Time-Domain Thermoreflectance. Adv. Mater..

[94] Zhu J., Tang D., Wang W., Liu J., Holub K.W., Yang R. (2010). Ultrafast thermoreflectance techniques for measuring thermal conductivity and interface thermal conductance of thin films. J. Appl. Phys..

[95] Raman C.V., Krishnan K.S. (1928). A New Type of Secondary Radiation. Nature.

[96] Butler H.J., Ashton L., Bird B., Cinque G., Curtis K., Dorney J., Esmonde-White K., Fullwood N.J., Gardner B., Martin-Hirsch P.L. (2016). Using Raman spectroscopy to characterize biological materials. Nat. Protoc..

[97] Ember K.J.I., Hoeve M.A., McAughtrie S.L., Bergholt M.S., Dwyer B.J., Stevens M.M., Faulds K., Forbes S.J., Campbell C.J. (2017). Raman spectroscopy and regenerative medicine: A review. NPJ Regen. Med..

[98] Cai Y., Lan J., Zhang G., Zhang Y.-W. (2014). Lattice vibrational modes and phonon thermal conductivity of monolayer MoS_2_. Phys. Rev. B.

[99] Tongay S., Zhou J., Ataca C., Lo K., Matthews T.S., Li J., Grossman J.C., Wu J. (2012). Thermally driven crossover from indirect toward direct bandgap in 2D semiconductors: MoSe_2_ versus MoS_2_. Nano Lett..

[100] Gordon K.C., McGoverin C.M. (2011). Raman mapping of pharmaceuticals. Int. J. Pharm..

[101] Xu H., Wu X., Li X., Luo C., Liang F., Orignac E., Zhang J., Chu J. (2018). Properties of graphene-metal contacts probed by Raman spectroscopy. Carbon.

[102] Li L., Liang X., Xu T., Xu F., Dong W. (2018). Rapid Detection of Six Glucocorticoids Added Illegally to Dietary Supplements by Combining TLC with Spot-Concentrated Raman Scattering. Molecules.

[103] Chen L.-C., Cao Z.-Y., Yu H., Jiang B.-B., Su L., Shi X., Chen L.-D., Chen X.-J. (2018). Phonon anharmonicity in thermoelectric palladium sulfide by Raman spectroscopy. Appl. Phys. Lett..

[104] Wang K., Li S., Petersen M., Wang S., Lu X. (2018). Detection and Characterization of Antibiotic-Resistant Bacteria Using Surface-Enhanced Raman Spectroscopy. Nanomaterials.

[105] Wu J., Wang P., Wang F., Fang Y. (2018). Investigation of the Microstructures of Graphene Quantum Dots (GQDs) by Surface-Enhanced Raman Spectroscopy. Nanomaterials.

[106] Wang J., Qiao W., Mu X. (2018). Au Tip-Enhanced Raman Spectroscopy for Catalysis. Appl. Sci..

[107] Gorbachev R.V., Riaz I., Nair R.R., Jalil R., Britnell L., Belle B.D., Hill E.W., Novoselov K.S., Watanabe K., Taniguchi T. (2011). Hunting for Monolayer Boron Nitride: Optical and Raman Signatures. Small.

[108] Zhou H., Zhu J., Liu Z., Yan Z., Fan X., Lin J., Wang G., Yan Q., Yu T., Ajayan P.M. (2014). High thermal conductivity of suspended few-layer hexagonal boron nitride sheets. Nano Res..

[109] Luo Z., Maassen J., Deng Y., Du Y., Garrelts R.P., Lundstrom M.S., Ye P.D., Xu X. (2015). Anisotropic in-plane thermal conductivity observed in few-layer black phosphorus. Nat. Commun..

[110] Yan R., Simpson J.R., Bertolazzi S., Brivio J., Watson M., Wu X., Kis A., Luo T., Hight Walker A.R., Xing H.G. (2014). Thermal Conductivity of Monolayer Molybdenum Disulfide Obtained from Temperature-Dependent Raman Spectroscopy. ACS Nano.

[111] Falkovsky L.A. (2008). Optical properties of graphene. J. Phys. Conf. Ser..

[112] Lee C., Wei X., Kysar J.W., Hone J. (2008). Measurement of the Elastic Properties and Intrinsic Strength of Monolayer Graphene. Science.

[113] Geim A.K. (2009). Graphene: Status and Prospects. Science.

[114] Sierra J.F., Neumann I., Cuppens J., Raes B., Costache M.V., Valenzuela S.O. (2018). Thermoelectric spin voltage in graphene. Nat. Nanotechnol..

[115] Hossain M.S., Huynh D.H., Jiang L., Rahman S., Nguyen P.D., Al-Dirini F., Hossain F., Bahk J.H., Skafidas E. (2018). Investigating enhanced thermoelectric performance of graphene-based nano-structures. Nanoscale.

[116] Li T., Pickel A.D., Yao Y., Chen Y., Zeng Y., Lacey S.D., Li Y., Wang Y., Dai J., Wang Y. (2018). Thermoelectric properties and performance of flexible reduced graphene oxide films up to 3000 K. Nat. Energy.

[117] Lim G., Kihm K.D., Kim H.G., Lee W., Lee W., Pyun K.R., Cheon S., Lee P., Min J.Y., Ko S.H. (2018). Enhanced Thermoelectric Conversion Efficiency of CVD Graphene with Reduced Grain Sizes. Nanomaterials (Basel).

[118] Qin D., Liu Y., Meng X., Cui B., Qi Y., Cai W., Sui J. (2018). Graphene-enhanced thermoelectric properties of P-type skutterudites. Chin. Phys. B.

[119] Li M., Cortie D.L., Liu J., Yu D., Islam S.M.K.N., Zhao L., Mitchell D.R.G., Mole R.A., Cortie M.B., Dou S. (2018). Ultra-high thermoelectric performance in graphene incorporated Cu_2_Se: Role of mismatching phonon modes. Nano Energy.

[120] Amollo T.A., Mola G.T., Kirui M.S.K., Nyamori V.O. (2017). Graphene for Thermoelectric Applications: Prospects and Challenges. Crit. Rev. Solid State Mater. Sci..

[121] Wang N., Li H., Ding C., Shi L., Jia H., Ren Z., Zhao Z. (2018). A Double-Voltage-Controlled Effective Thermal Conductivity Model of Graphene for Thermoelectric Cooling. IEEE Trans. Electron Devices.

[122] Mingo N., Broido D.A. (2005). Carbon Nanotube Ballistic Thermal Conductance and Its Limits. Phys. Rev. Lett..

[123] Nika D.L., Pokatilov E.P., Askerov A.S., Balandin A.A. (2009). Phonon thermal conduction in graphene: Role of Umklapp and edge roughness scattering. Phys. Rev. B.

[124] Calizo I., Balandin A.A., Bao W., Miao F., Lau C.N. (2007). Temperature Dependence of the Raman Spectra of Graphene and Graphene Multilayers. Nano Lett..

[125] Ghosh S., Calizo I., Teweldebrhan D., Pokatilov E.P., Nika D.L., Balandin A.A., Bao W., Miao F., Lau C.N. (2008). Extremely high thermal conductivity of graphene: Prospects for thermal management applications in nanoelectronic circuits. Appl. Phys. Lett..

[126] Ghosh S., Bao W., Nika D.L., Subrina S., Pokatilov E.P., Lau C.N., Balandin A.A. (2010). Dimensional crossover of thermal transport in few-layer graphene. Nat. Mater..

[127] Nika D.L., Ghosh S., Pokatilov E.P., Balandin A.A. (2009). Lattice thermal conductivity of graphene flakes: Comparison with bulk graphite. Appl. Phys. Lett..

[128] Balandin A.A. (2011). Thermal properties of graphene and nanostructured carbon materials. Nat. Mater..

[129] Chen S., Moore A.L., Cai W., Suk J.W., An J., Mishra C., Amos C., Magnuson C.W., Kang J., Shi L. (2011). Raman Measurements of Thermal Transport in Suspended Monolayer Graphene of Variable Sizes in Vacuum and Gaseous Environments. ACS Nano.

[130] Lee J.-U., Yoon D., Kim H., Lee S.W., Cheong H. (2011). Thermal conductivity of suspended pristine graphene measured by Raman spectroscopy. Phys. Rev. B.

[131] Dobrzhinetskaya L.F., Wirth R., Yang J., Green H.W., Hutcheon I.D., Weber P.K., Grew E.S. (2014). Qingsongite, natural cubic boron nitride: The first boron mineral from the Earth’s mantle. Am. Mineral..

[132] Weng Q., Wang X., Wang X., Bando Y., Golberg D. (2016). Functionalized hexagonal boron nitride nanomaterials: Emerging properties and applications. Chem. Soc. Rev..

[133] Li L.H., Chen Y. (2016). Atomically Thin Boron Nitride: Unique Properties and Applications. Adv. Funct. Mater..

[134] Lindsay L., Broido D.A. (2011). Enhanced thermal conductivity and isotope effect in single-layer hexagonal boron nitride. Phys. Rev. B.

[135] Tao O., Yuanping C., Yuee X., Kaike Y., Zhigang B., Jianxin Z. (2010). Thermal transport in hexagonal boron nitride nanoribbons. Nanotechnology.

[136] Wang Y., Xu N., Li D., Zhu J. (2017). Thermal Properties of Two Dimensional Layered Materials. Adv. Funct. Mater..

[137] Wang C., Guo J., Dong L., Aiyiti A., Xu X., Li B. (2016). Superior thermal conductivity in suspended bilayer hexagonal boron nitride. Sci. Rep..

[138] Jo I., Pettes M.T., Kim J., Watanabe K., Taniguchi T., Yao Z., Shi L. (2013). Thermal Conductivity and Phonon Transport in Suspended Few-Layer Hexagonal Boron Nitride. Nano Lett..

[139] Li L., Yu Y., Ye G.J., Ge Q., Ou X., Wu H., Feng D., Chen X.H., Zhang Y. (2014). Black phosphorus field-effect transistors. Nat. Nanotechnol..

[140] Engel M., Steiner M., Avouris P. (2014). Black Phosphorus Photodetector for Multispectral, High-Resolution Imaging. Nano Lett..

[141] Xia F., Wang H., Jia Y. (2014). Rediscovering black phosphorus as an anisotropic layered material for optoelectronics and electronics. Nat. Commun..

[142] Zhang G., Huang S., Chaves A., Song C., Özçelik V.O., Low T., Yan H. (2017). Infrared fingerprints of few-layer black phosphorus. Nat. Commun..

[143] Buscema M., Groenendijk D.J., Blanter S.I., Steele G.A., van der Zant H.S.J., Castellanos-Gomez A. (2014). Fast and Broadband Photoresponse of Few-Layer Black Phosphorus Field-Effect Transistors. Nano Lett..

[144] Yuan H., Liu X., Afshinmanesh F., Li W., Xu G., Sun J., Lian B., Curto A.G., Ye G., Hikita Y. (2015). Polarization-sensitive broadband photodetector using a black phosphorus vertical p–n junction. Nat. Nanotechnol..

[145] Wu P., Appenzeller J. High Performance Complementary Black Phosphorus FETs and Inverter Circuits Operating at Record-Low VDDdown to 0.2V. Proceedings of the 2018 76th Device Research Conference (DRC).

[146] Zhang X., Xie H., Liu Z., Tan C., Luo Z., Li H., Lin J., Sun L., Chen W., Xu Z. (2015). Black Phosphorus Quantum Dots. Angew. Chem. Int. Ed..

[147] Liu Y., Weiss N.O., Duan X., Cheng H.-C., Huang Y., Duan X. (2016). Van der Waals heterostructures and devices. Nat. Rev. Mater..

[148] Ong Z.-Y., Cai Y., Zhang G., Zhang Y.-W. (2014). Strong Thermal Transport Anisotropy and Strain Modulation in Single-Layer Phosphorene. J. Phys. Chem. C.

[149] Peng X., Copple A., Wei Q. (2014). Edge effects on the electronic properties of phosphorene nanoribbons. J. Appl. Phys..

[150] Rodin A.S., Carvalho A., Castro Neto A.H. (2014). Strain-Induced Gap Modification in Black Phosphorus. Phys. Rev. Lett..

[151] Konabe S., Kawabata S., Yamamoto T. (2016). Thermoelectric properties of bilayer phosphorene under tensile strain. Surf. Interface Anal..

[152] Luo Z.-Z., Zhang Y., Zhang C., Tan H.T., Li Z., Abutaha A., Wu X.-L., Xiong Q., Khor K.A., Hippalgaonkar K. (2016). Multifunctional 0D–2D Ni_2_P Nanocrystals–Black Phosphorus Heterostructure. Adv. Energy Mater..

[153] Saito Y., Iizuka T., Koretsune T., Arita R., Shimizu S., Iwasa Y. (2016). Gate-Tuned Thermoelectric Power in Black Phosphorus. Nano Lett..

[154] Zhang J., Liu H.J., Cheng L., Wei J., Liang J.H., Fan D.D., Jiang P.H., Sun L., Shi J. (2016). High thermoelectric performance can be achieved in black phosphorus. J. Mater. Chem. C.

[155] Flores E., Ares J.R., Castellanos-Gomez A., Barawi M., Ferrer I.J., Sánchez C. (2015). Thermoelectric power of bulk black-phosphorus. Appl. Phys. Lett..

[156] Pang J., Bachmatiuk A., Yin Y., Trzebicka B., Zhao L., Fu L., Mendes R.G., Gemming T., Liu Z., Rummeli M.H. (2018). Applications of Phosphorene and Black Phosphorus in Energy Conversion and Storage Devices. Adv. Energy Mater..

[157] Zhang Y., Zheng Y., Rui K., Hng H.H., Hippalgaonkar K., Xu J., Sun W., Zhu J., Yan Q., Huang W. (2017). 2D Black Phosphorus for Energy Storage and Thermoelectric Applications. Small.

[158] Qin G., Yan Q.B., Qin Z., Yue S.Y., Cui H.J., Zheng Q.R., Su G. (2014). Hinge-like structure induced unusual properties of black phosphorus and new strategies to improve the thermoelectric performance. Sci. Rep..

[159] Fei R., Faghaninia A., Soklaski R., Yan J.-A., Lo C., Yang L. (2014). Enhanced Thermoelectric Efficiency via Orthogonal Electrical and Thermal Conductances in Phosphorene. Nano Lett..

[160] Zhang J., Liu H.J., Cheng L., Wei J., Liang J.H., Fan D.D., Shi J., Tang X.F., Zhang Q.J. (2014). Phosphorene nanoribbon as a promising candidate for thermoelectric applications. Sci. Rep..

[161] Liu H., Neal A.T., Zhu Z., Luo Z., Xu X., Tománek D., Ye P.D. (2014). Phosphorene: An Unexplored 2D Semiconductor with a High Hole Mobility. ACS Nano.

[162] Jang H., Wood J.D., Ryder C.R., Hersam M.C., Cahill D.G. (2015). Anisotropic Thermal Conductivity of Exfoliated Black Phosphorus. Adv. Mater..

[163] An C.J., Kang Y.H., Lee C., Cho S.Y. (2018). Preparation of Highly Stable Black Phosphorus by Gold Decoration for High-Performance Thermoelectric Generators. Adv. Funct. Mater..

[164] Andres C.-G., Leonardo V., Elsa P., Joshua O.I., Narasimha-Acharya K.L., Sofya I.B., Dirk J.G., Michele B., Gary A.S., Alvarez J.V. (2014). Isolation and characterization of few-layer black phosphorus. 2D Mater..

[165] Ribeiro H.B., Pimenta M.A., de Matos C.J.S. (2018). Raman spectroscopy in black phosphorus. J. Raman Spectrosc..

[166] Lee S., Yang F., Suh J., Yang S., Lee Y., Li G., Sung Choe H., Suslu A., Chen Y., Ko C. (2015). Anisotropic in-plane thermal conductivity of black phosphorus nanoribbons at temperatures higher than 100 K. Nat. Commun..

[167] Wu J., Mao N., Xie L., Xu H., Zhang J. (2015). Identifying the Crystalline Orientation of Black Phosphorus Using Angle-Resolved Polarized Raman Spectroscopy. Angew. Chem. Int. Ed..

[168] Ribeiro H.B., Pimenta M.A., de Matos C.J.S., Moreira R.L., Rodin A.S., Zapata J.D., de Souza E.A.T., Castro Neto A.H. (2015). Unusual Angular Dependence of the Raman Response in Black Phosphorus. ACS Nano.

[169] Wang Q.H., Kalantar-Zadeh K., Kis A., Coleman J.N., Strano M.S. (2012). Electronics and optoelectronics of two-dimensional transition metal dichalcogenides. Nat. Nanotechnol..

[170] Chhowalla M., Shin H.S., Eda G., Li L.-J., Loh K.P., Zhang H. (2013). The chemistry of two-dimensional layered transition metal dichalcogenide nanosheets. Nat. Chem..

[171] Jariwala D., Sangwan V.K., Lauhon L.J., Marks T.J., Hersam M.C. (2014). Emerging Device Applications for Semiconducting Two-Dimensional Transition Metal Dichalcogenides. ACS Nano.

[172] Manzeli S., Ovchinnikov D., Pasquier D., Yazyev O.V., Kis A. (2017). 2D transition metal dichalcogenides. Nat. Rev. Mater..

[173] Choi W., Choudhary N., Han G.H., Park J., Akinwande D., Lee Y.H. (2017). Recent development of two-dimensional transition metal dichalcogenides and their applications. Mater. Today.

[174] Zhang H., Chhowalla M., Liu Z. (2018). 2D nanomaterials: Graphene and transition metal dichalcogenides. Chem. Soc. Rev..

[175] Zhang X., Lai Z., Ma Q., Zhang H. (2018). Novel structured transition metal dichalcogenide nanosheets. Chem. Soc. Rev..

[176] Wan C., Gu X., Dang F., Itoh T., Wang Y., Sasaki H., Kondo M., Koga K., Yabuki K., Snyder G.J. (2015). Flexible N-type thermoelectric materials by organic intercalation of layered transition metal dichalcogenide TiS_2_. Nat. Mater..

[177] Huang H., Cui Y., Li Q., Dun C., Zhou W., Huang W., Chen L., Hewitt C.A., Carroll D.L. (2016). Metallic 1T phase MoS_2_ nanosheets for high-performance thermoelectric energy harvesting. Nano Energy.

[178] Lee C., Hong J., Whangbo M.-H., Shim J.H. (2013). Enhancing the Thermoelectric Properties of Layered Transition-Metal Dichalcogenides 2H-MQ_2_ (M = Mo, W; Q = S, Se, Te) by Layer Mixing: Density Functional Investigation. Chem. Mater..

[179] Lopez-Sanchez O., Lembke D., Kayci M., Radenovic A., Kis A. (2013). Ultrasensitive photodetectors based on monolayer MoS_2_. Nat. Nanotechnol..

[180] Chi Z.-H., Zhao X.-M., Zhang H., Goncharov A.F., Lobanov S.S., Kagayama T., Sakata M., Chen X.-J. (2014). Pressure-Induced Metallization of Molybdenum Disulfide. Phys. Rev. Lett..

[181] Fan D.D., Liu H.J., Cheng L., Jiang P.H., Shi J., Tang X.F. (2014). MoS_2_ nanoribbons as promising thermoelectric materials. Appl. Phys. Lett..

[182] Sahoo S., Gaur A.P.S., Ahmadi M., Guinel M.J.F., Katiyar R.S. (2013). Temperature-Dependent Raman Studies and Thermal Conductivity of Few-Layer MoS_2_. J. Phys. Chem. C.

[183] Yalon E., Aslan O.B., Smithe K.K.H., McClellan C.J., Suryavanshi S.V., Xiong F., Sood A., Neumann C.M., Xu X., Goodson K.E. (2017). Temperature-Dependent Thermal Boundary Conductance of Monolayer MoS2 by Raman Thermometry. ACS Appl. Mater. Interfaces.

[184] Zhang X., Sun D., Li Y., Lee G.H., Cui X., Chenet D., You Y., Heinz T.F., Hone J.C. (2015). Measurement of Lateral and Interfacial Thermal Conductivity of Single- and Bilayer MoS_2_ and MoSe_2_ Using Refined Optothermal Raman Technique. ACS Appl. Mater. Interfaces.

[185] Chen L.-C., Yu H., Pang H.-J., Jiang B.-B., Su L., Shi X., Chen L.-D., Chen X.-J. (2018). Pressure-induced enhancement of thermoelectric performance in palladium sulfide. Mater. Today Phys..

[186] Yu H., Chen L.-C., Pang H.-J., Qin X.-Y., Qiu P.-F., Shi X., Chen L.-D., Chen X.-J. (2018). Large enhancement of thermoelectric performance in CuInTe_2_ upon compression. Mater. Today Phys..

[187] Zhang Y., Hao S., Zhao L.-D., Wolverton C., Zeng Z. (2016). Pressure induced thermoelectric enhancement in SnSe crystals. J. Mater. Chem..

